# Oral health knowledge and oral health related quality of life of older adults

**DOI:** 10.1002/cre2.350

**Published:** 2020-11-17

**Authors:** So Ran Kwon, Shirley Lee, Udochukwu Oyoyo, Seth Wiafe, Samantha De Guia, Caitlin Pedersen, Kelsey Martinez, Joscelyn Rivas, Daniela Chavez, Tom Rogers

**Affiliations:** ^1^ Center for Dental Research Loma Linda University School of Dentistry Loma Linda California USA; ^2^ Department of Dental Hygiene Loma Linda University School of Dentistry Loma Linda California USA; ^3^ Loma Linda University School of Public Health Loma Linda California USA

**Keywords:** education, OHIP, older adults, oral health knowledge, oral health related quality of life

## Abstract

**Objective:**

To assess the relationship between oral health knowledge and oral health related quality of life among older adults with different ethnicities living in San Bernardino County, California. There is a gap in oral health knowledge (OHK) and how it relates to perceived oral health related quality of life. Thus, there is a need to assess OHK as a component of oral health literacy and identify areas in which knowledge gaps exit to develop educational strategies that address the need of the elderly population.

**Materials and Methods:**

The study was a cross‐sectional study that included adults 65 years and older using a validated “Comprehensive Measure of Oral Health Knowledge” (CMOHK) and an “Oral Health Profile Index” (OHIP‐14). Odds ratios were conducted to determine the factors associated with OHK.

**Results:**

Mean OHK score were 16.8, 14.6, and 8.9 for Caucasian, Asian, and Hispanics, respectively. “Poor” OHK was significantly associated with participants over the age of 75 years (OR = 1.9; 95% CI: 1.15–3.16), high school education or less (OR = 10.8; 95% CI: 5.92–19.84), minority ethnicity (OR = 7.3; 95% CI: 4.27–12.61), income less than $25,000 (OR = 10.7; 95% CI: 5.92–19.26), and reading ability less than “Excellent” (OR = 7.27; 95% CI: 4.35–12.14). Mean OHIP‐Severity scores were 7.4, 12.5, and 24.4 for Caucasian, Asian, and Hispanics, respectively. Respondents with Poor OHK were 5.17 times more likely to be identified with high levels of severity (Severity >10).

**Conclusion:**

It is imperative to develop communication strategies to inform older adults on oral health knowledge that provide equal opportunities for all ethnicities.

## 1 INTRODUCTION

Every day 10,000 Americans turn 65 years of age and by 2030, one out of five Americans will be over the age of 65 (Federal Interagency Forum on Aging‐Related Statistics, 2012). Dental and scientific advances offer new innovations for the improvement of oral health for our society. Yet a disproportionate burden of poor oral health continues among many underserved minority groups and elderly people (Lazarchik & Haywood, 2010). Currently, seven out of ten of older adults have periodontal disease, one out of five have untreated tooth decay, and one out of four have lost some or all of their teeth (Eke et al., 2012; No Authors, 2001). Optimal oral health can be achieved by, 1) strong commitment to maintain oral care that can be fostered by effective communication strategies on oral health information and programs; and 2) providing adequate ongoing professional care that can be accessed readily.

Oral health can be measured objectively by means of oral examinations by oral health professionals and also subjectively as reported by the individual. The use of subjective measures in evaluating oral health is well‐established (Lee, Shieh, et al., 2007). There is a vast evidence showing that the perceived dental condition is closely related to the individual's oral health‐related quality of life (OHRQoL), and may have a greater impact than the actual presentation of the clinical condition (Brennan & Spencer, 2006). The United States (US) Surgeon General's report on Oral Health in America emphasizes the importance of OHRQoL, and its improvement on a population‐level is defined as a goal (Department of Health and Human Services, 2000). The validated short form of oral health index profile (OHIP‐14) is used to quantify patient outcome experiences, monitor oral health status on national level, and identify dental public health goals (Sanders et al., 2009).

Oral health literacy is the premise for better oral health and defined as “the degree to which individuals have the capacity to obtain, process and understand basic health information and services needed to make appropriate oral health decisions” (J Public Health Dent, 2005). Instruments that measure oral health literacy include, the REALD‐ 30 (Lee, Rozier, et al., 2007) and the TOFHLAiD (Gong et al., 2007). However, main shortcomings include that they primarily evaluate word recognition and reading comprehension, and fail to determine oral health knowledge. The Comprehensive Measure of Oral Health Knowledge (CMOHK) which was developed at the University of Maryland is a validated 23‐item questionnaire (Macek et al., 2010), that focuses on determining basic oral health knowledge. Upon completing the questionnaire, the individual's oral health knowledge can be assessed and categorized as good or poor.

Poor oral health literacy has been associated with inadequate oral health outcomes such as poor oral health status, dental neglect and sporadic dental attendance (Divaris et al., 2011; National Institute of Dental and Craniofacial Research, National Institute of Health, U.S. Public Health Service, Department of Health and Human Services, 2005; Wehmeyer et al., 2014). Effective communication strategies are based on appropriate understanding of the target population. However, there is a gap in oral health knowledge of the elderly population and how it relates to perceived oral health related quality of life. Thus, there is a need to assess the oral health knowledge as a component of oral health literacy and identify areas in which knowledge gaps exit to develop effective educational strategies that meet the needs of the elderly population.

The purpose of our study was to use a validated “Comprehensive Measure of Oral Health Knowledge (CMOHK)” and an “Oral Health Profile Index (OHIP‐14)” to assess the relationship between oral health knowledge and oral health related quality of life among older adults living in San Bernardino County, California. We hypothesized that there would be no meaningful difference in oral health related knowledge as measured with the Oral Health Profile Index (OHIP‐14) and oral health related quality of life OHRQoL among San Bernardino County racial/ethnic groups.

## 2 MATERIALS AND METHODS

An IRB application was approved by the Loma Linda University Institutional Review Board (IRB #5180323) for a cross‐sectional study that included adults 65 years and older living in San Bernardino County, California. Potential participants were contacted by a research team member at Loma Linda University School of Dentistry, local churches, and community centers and asked if they were 65 years and older and would fill‐out a survey. Participants could take their time while filling out the survey and were not required to fill‐out all questions. Participants received a complimentary toothbrush and toothpaste at the time of participation.

The questions were selected from two validated questionnaires (English and Spanish) and also specifically formulated for this study. The questionnaire included: demographic information, daily oral hygiene procedures, dental utilization, oral health knowledge, and oral health related quality of life. Questions were selected and utilized verbatim or modified from the Comprehensive Measure of Oral Health Knowledge (CMOHK), and Short form of the Oral Health Profile Index (OHIP‐14).

Participants had the option to fill‐out the questionnaire in English, Spanish, or a mix of English and Spanish. For data analysis, each question was scored as wrong or correct. Then the total number of correct answers was calculated. Originally knowledge scores were categorized into three categories: poor, fair, and good (Macek et al., 2010). We used a modified scoring system that better served our purpose, and knowledge scores were simply split into poor (0–14) or good (15–23) (McQuistan et al., 2014; Patino et al., 2018).

The short form of the Oral Health Impact Profile (OHIP‐14) index was used to evaluate OHRQoL (Slade, 1997). OHRQoL impacts were assessed by calculating ***Severity*** (cumulative OHIP‐14 score ranging from 0–56).

A sample size calculation was conducted in order to find an effect size of 0.2 (10% difference) in oral health knowledge between Caucasians and Hispanics. Based on the calculation, it was estimated that a minimum sample size of 60 per group should be obtained for 80% power, at an alpha level of 0.05.

Descriptive statistics were calculated for all dependent and independent variables. Independent variables were categorized into three domains: 1) demographics, 2) oral hygiene practices, and 3) dental utilization. Odds ratios were conducted to determine the factors associated with oral health knowledge and dental utilization within each domain and were reported with 95% confidence intervals with continuity corrections. The findings of descriptive analyses were reported as absolute frequencies or rates in the case of categorical variables, as medians in the case of quantitative variables with non‐parametric distributions, and as mean ± SD in the case of quantitative variables with normal distributions. Quantitative variables were compared among the study groups by using one‐way analysis of variance (ANOVA) or the Kruskal–Wallis test, as appropriate. Categorical variables were compared using the χ^2^ test. Correlations among Oral Health Knowledge and Oral Health Profile Index Severity were conducted with Pearson Correlation. Binary multivariate logistic regression analysis with odds ratios and 95% confidence intervals was performed to identify independent variables predicting oral health knowledge and severity of oral health. *p* values < 0.05 were considered statistically significant. All data were analyzed using SAS version 9.4 and R 3.6.2.

## 3 RESULTS

All surveys were collected and responses entered into a data spreadsheet. There were no limitations of skipped questions. Overall there were only two questions that were skipped by one participant. The skipped questions were counted as incorrect. Out of a total of 304 surveys 127 (41.8%) were filled out in the Spanish version. Despite the option to use both the Spanish and English version together, there were no mixed versions. The ethnicity distribution of older adults aged 65 years and above for our study compared to the US Census of San Bernardino County is summarized in Table 1. Overall our study reflected the ethnicity distribution of San Bernardino County, with higher percentages observed in the Hispanic and Asian group while lower percentages were observed for the Caucasian group compared to the US Census records.

**TABLE 1:** Ethnicity distribution of older adults 65 years and above for our study versus US Census of San Bernardino County (%)

Ethnicity	Our study	US census SBC
Caucasian	32.9	50.0
Hispanic	41.8	31.1
Asian	19.1	9.3
African‐American	5.3	7.7
Other	1.0	1.9

Other demographics, oral hygiene practices, and dental utilization are summarized in Table 2. Over two‐thirds (67.8%) of the study sample was between the ages of 65 and 75 years. More than half of the participants were female (57.9%) or had more than high school education (60.5%). Less than half of the participants earned less than $25,000 (37.8%) or reported excellent reading ability (40.8%). Over two‐thirds of the study sample brushed twice or more a day (70.4%) or used a manual toothbrush (69.7%). The majority of respondents received regular dental care (74.0%) while half had dental insurance (52.3%).

**TABLE 2:** Summary of demographics, oral hygiene care practices, and dental utilization of respondents (*N* = 304)

Demographics	
**Age range**	Percentage
65–70	38.16
71–75	29.61
76–80	19.08
81–85	8.88
86 and above	4.28
**Gender**	
Male	42.11
Female	57.89
**Education**	
High school or less	39.47
High school or more	60.53
**Income**	
>$25,000	45.07
<$25,000	37.83
Not wish to respond	17.11
**Reading ability**	
Excellent	40.79
Good	26.32
OK	17.76
Poor	6.58
Terrible	5.92
**Oral hygiene care practices**	
**Brushing habits**	
Rarely	7.57
Once/day	22.04
Twice/day	52.96
More than twice/day	17.43
**Type of toothbrush**	
Manual	69.74
Powered	14.47
Both	12.17
Other	3.29
**Auxiliaries used**	
Mouthrinse	55.59
Floss	44.74
Interdental brush	12.50
Waterfloss	7.89
Other	Denture brush, toothpick, floss thread, tongue scraper
**Toothbrush holding difficulties**	
Great difficulty	1.97
Moderate difficulty	6.25
No difficulty	91.78
**Awareness on number of teeth left in mouth**	
Aware	52.3
Do not know	47.70
**Dental utilization**	
**Dental care history**	
Regular care	74.01
Sporadic care	25.99
**Dental insurance**	
Yes	52.30
No	43.42
Do not know	4.28

The mean OHK score was 13. Scores ranged from 0 through 23 points, with two participants having all questions answered incorrectly and one participant having all 23 responses correct. Overall the mean OHK score and SD were 16.8 (4.0), 14.6 (4.7), and 8.9 (4.7) for Caucasian, Asian, and Hispanics, respectively. There was a statistically significant difference among the three ethnicity groups (*p* < 0.05). Approximately 56.9% of participants were identified as having “Poor” oral health knowledge (0–14 points). When looking at each question individually as summarized in Table 3, most participants correctly answered questions that pertained to general dental knowledge (except for questions on teeth numbers), dental treatment, and presentation of oral diseases. However, respondents were less likely to correctly answer questions that pertained to children's oral health, periodontal disease, and oral cancer. Higher percentage of correct responses for each question by ethnicity was generally in the order of Caucasian, Asian, and Hispanics.

**TABLE 3:** Percentage of correct responses to CMOHK questions by ethnicity

Question	Category	All	A	C	H
1. What is another name for the roof of your mouth?	General knowledge‐Terminology	73	67	79	71
2. This picture shows the inside of a person's mouth. The arrow points to something hanging from the back of the throat.	General knowledge‐Terminology	61	55	65	62
3. How many baby teeth does a child usually get?	General knowledge‐Teeth numbers	21	28	24	10
4. How many permanent teeth does an adult usually get?	General knowledge‐Teeth numbers	46	55	71	18
5. How old are children when they get their first adult tooth?	General knowledge‐Teeth numbers	40	40	60	24
6. As you understand it, what is the main purpose of braces?	Treatment‐Ortho	85	88	100	69
7. As you understand it, what is the main purpose of adding fluoride to the public drinking water?	Treatment‐Fluoride	53	62	79	28
8. As you understand it, what is the main purpose of dental implants?	Treatment‐Implant	79	98	98	53
9. This picture shows different parts of a tooth. To what part of the tooth is the arrow pointing?	General knowledge‐Terminology	61	71	89	32
10. According to the American Dental Association, how often should adults who have their own teeth visit the dentist?	Treatment‐Visits	62	74	72	45
11. In order to prevent tooth decay, people should avoid food with a lot of which of the following?	Prevention‐Decay	80	86	89	67
12. What is the main reason infants should not be put to bed with a bottle that contains fruit juice?	Risk‐Decay	46	59	76	17
13. What is the best way a person can prevent tooth decay at home?	Prevention‐Decay	77	91	93	56
14. When a person has a small cavity, how does the dentist usually treat it?	Treatment‐Restorative	72	79	99	46
15. When a person has a large cavity, sometimes he or she needs a root canal. Which of the following describes what a root canal is?	Treatment‐Endo	60	69	84	36
16. This picture shows the inside of a child's mouth. What do you think is wrong	Presentation‐Rampant caries	57	78	79	27
17. This picture shows some gums that are puffy and red. What do you think this condition is called?	Presentation‐Perio disease	59	57	71	49
18. Which of the following behaviors may cause periodontal disease?	Risk‐Perio disease	46	47	56	36
19. Which of the following is the best way to remove tartar from a person's teeth?	Treatment‐Cleaning	59	66	71	46
20. This picture shows some teeth with receding gums. What do you think this condition is called?	Presentation‐Perio disease	55	71	81	21
21. Periodontal disease is more likely to occur in people with which of the following conditions?	Risk‐Perio disease	44	50	46	39
22. What is the most common sign of cancer inside the mouth?	Presentation‐Cancer	37	47	61	14
23. Which of the following groups is most likely to get cancer inside their mouth?	Risk‐Cancer	27	22	34	25

Abbreviations: A, Asian; C, Caucasian; H, Hispanic.

“Poor” oral health knowledge was significantly associated with participants over the age of 75 years (OR = 1.9; 95% CI: 1.15–3.16), high school education or less (OR = 10.8; 95% CI: 5.92–19.84), minority ethnicity (OR = 7.3; 95% CI: 4.27–12.61), income less than $25,000 (OR = 10.7; 95% CI: 5.92–19.26), and reading ability less than “Excellent” (OR = 7.27; 95% CI: 4.35–12.14). The ethnicity association with “Poor” oral health knowledge is summarized in Table 4. Hispanic participants were nearly 25 times more likely to be identified as “Poor” oral health knowledge as compared to Caucasians. Asians were over 2.5 times more likely to be identified as ‘Poor” OHK. When modeling for variables associated with poor oral health knowledge, ethnicity was the strongest factor followed by “How often they brush their teeth ‘(OR=5.73; 95% CI: 1.33‐24.75)’ and ‘Reading Quality’ (OR = 2.60; 95% CI: 1.36–4.95).”

**TABLE 4:** Ethnicity association with “Poor” oral health knowledge

	Frequency	Valid percent	df	Sig.	Odds ratio	95% Confidence interval	
						Lower	Upper
Caucasian	100	32.9					
Hispanic	127	41.8	1	0	24.96	12.06	51.65
Asian	58	19.1	1	0.009	2.48	1.25	4.9

**FIGURE 1:**
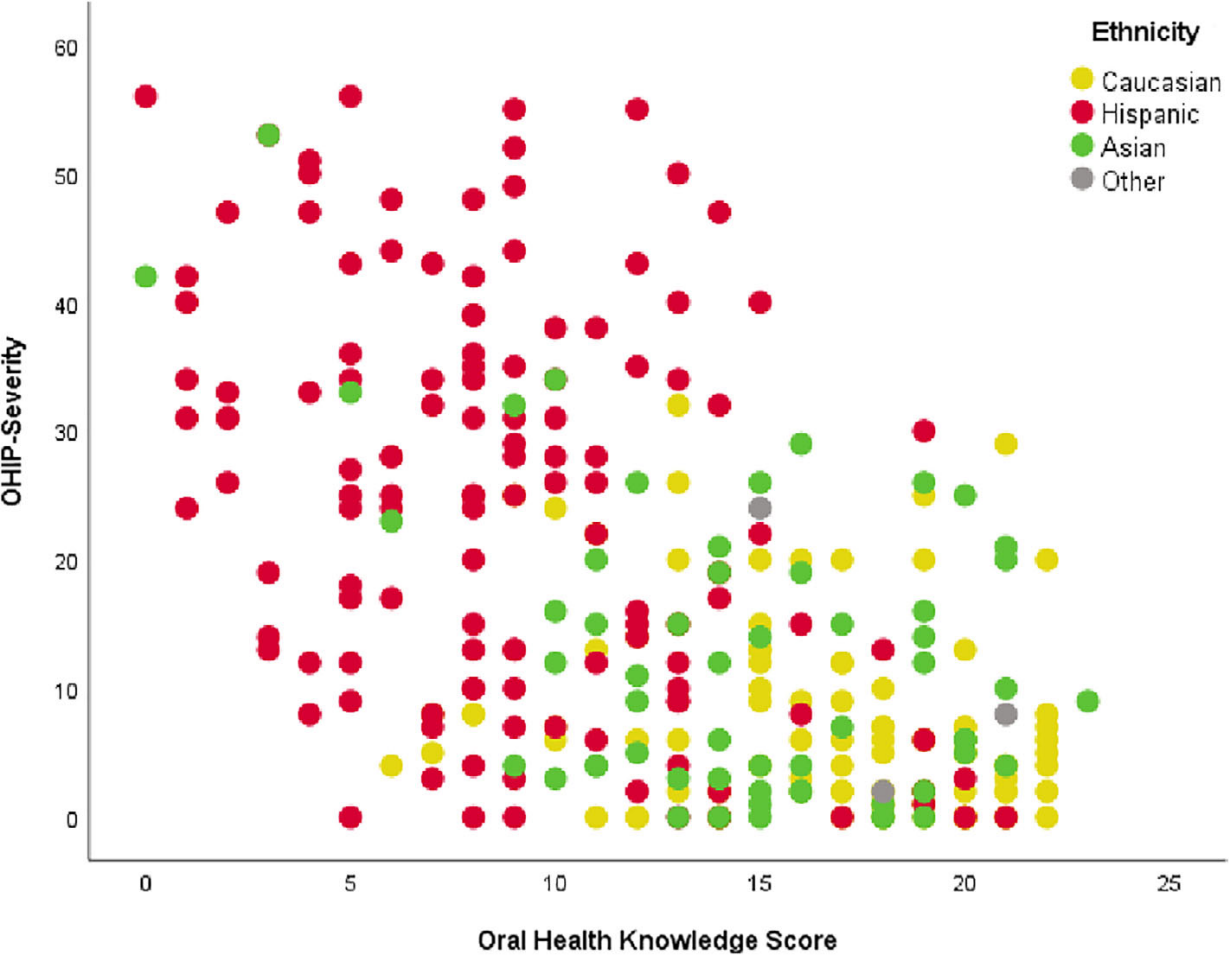
Scatterplot of relationship between oral health knowledge and oral health profile index severity by ethnicity groups

OHRQoL was assessed using a 5‐point scale from 0 noting “never” to 4 noting “very often.” The severity score ranged from 0 to 56 with a mean score of 16. Overall the mean OHIP severity score and SD were 7.4 (7.6), 12.5 (11.8), and 24.4 (15.9) for Caucasian, Asian, and Hispanics, respectively. There was a statistically significant difference among the three ethnicity groups (*p* < 0.001) with Caucasians having the lowest and Hispanics the highest severity. Most participants responded never or hardly ever to the question pertaining to inability to function because of problems with their teeth or mouth while most participants reported occasionally experiencing painful aching in the mouth, discomfort while eating food, and feeling self‐conscious because of their teeth and mouth. Severity was significantly higher (*p* < .001) for participants with “Poor” oral health knowledge (Median = 19; IQR: 8–33) as compared to participants with “Good” oral health knowledge (Median = 6; IQR: 2–12). Respondents with Poor OHK were 5.17 times more likely to be identified with high levels of severity (Severity >10). The strongest factor associated with severity (>10) was Education of High School or less (OR = 3.93; 95% CI: 1.95–7.91). The relationship between Oral Health Knowledge and Oral Health Profile Index Severity by ethnicity is illustrated in Figure 1. as a scatterplot. Overall higher OHK scores were associated with lower OHIP Severity scores (R = −.563, *p* < .001). However, this relationship was only statistically significant among Caucasian (R = −.272, *p* = .006) and Hispanic participants (R = −.383, *p* < .001), but not among Asian (R = −.463, *p* = .060) and other participants (R = −.703, *p* = .503).

## 4 DISCUSSION

Older adults are a vulnerable population that can suffer disproportionately from a variety of diseases. Although often recognized as a single cohort, older adults are a diverse population, spanning 35 plus years. Generally, adults between 65 to 75 years are defined as the “young old,” 75 to 85 years as the “old,” and 85 years and above as “old old.” Within each category, older adults have distinct cultural, psychological, educational, social, economic, dietary and chronological experiences that define their life and way of thinking (Ettinger & Beck, 1984; Ettinger & Mulligan, 1999).

Our study evaluated oral health knowledge and oral health related quality of life of older adults among different ethnicities living in San Bernardino County, California. Over two thirds of participants belonged to the “young old” category and the majority were living in the community. Thus, the main focus of our study was related to functionally independent older adults (Ettinger & Beck, 1984; Ettinger & Mulligan, 1999). It is important to note that compared with previous generations, the current older adults are more likely to retain their teeth and consequently more likely to experience periodontal disease, root caries, and oral cancer (Ettinger & Beck, 1984; Ettinger & Mulligan, 1999; Qualtrough & Mannocci, 2011). This underscores the importance of proper oral health knowledge and ways to prevent and treat common oral diseases. However, based on our results, there was a lack of knowledge on questions related to children's oral health, periodontal disease, and oral cancer. This is in accordance with another study that evaluated oral health knowledge among elderly patients at the University of Iowa College of Dentistry (McQuistan et al., 2014). Although knowledge on children's oral health may not be directly related to oral care of older adults themselves, it is still important as a significant number of older adults (6%) live with their grandchildren and provide some type of child care that may affect oral care instructions that they provide to them (Luo et al., 2012).

However, while approximately six out of ten participants in our study were identified as having “Poor” OHK only 34% of participants in the Iowa study received a poor score (McQuistan et al., 2014). This discordance may be attributed to two distinct differences in the study population. First, our study included different ethnicities including Caucasians, Hispanics, and Asians while the race of participants in the Iowa study was mainly White. Another difference may be that participants in the Iowa study were patients seeking dental care at the institution. Our study included participants contacted at Loma Linda University School of Dentistry, local churches, and community centers who were not necessarily patients coming for dental treatment.

Poor OHK was associated with increasing age, education, ethnicity, income, and reading ability. When modeling for variables associated with poor oral health knowledge, ethnicity was the strongest factor with Hispanic participants nearly 25 times more likely to be identified as “Poor” oral health knowledge as compared to Caucasians. Based on our results we rejected our null hypothesis, since there was a difference in OHK among different ethnicities. The concern of poor OHK among Hispanics was also pointed out by a study that evaluated the OHK specifically in the Hispanic population. Despite the lower mean age of 38 years, the mean OHK in that study was still low at 14 (Patino et al., 2018). The knowledge gap existed in areas of children's oral health, periodontal disease, and oral cancer which was in accordance with our study.

An interesting finding of our study that has not been noted in other literature was that less than half of participants were not knowledgeable about the number of permanent teeth in adults. Tooth loss is an important indicator of oral health and the number of teeth affect the ability to chew, speak, and socialize. The California Oral Health Plan 2018–2028 (California Department of Public Health, 2018), outlines an objective to reduce the proportion of adults who have ever had a permanent tooth extracted because of dental caries or periodontal disease. Based on goal setting theories, to improve health behavior change and maintenance interventions, it is important to target specific goals leading to higher performance when compared with no goals or vague, nonquantitative goals such as preserve as many teeth as possible (Strecher et al., 1995). Therefore, from a knowledge perspective, it may be highly desirable to address the gap in general knowledge on the number of permanent teeth in adults. This knowledge may impart a sense of value for each tooth and also recognize the importance for maximum preservation of teeth throughout one's lifetime.

The short version OHIP‐14 is a commonly used tool to assess the negative impacts of oral problems on the perceived quality of life of individuals. The mean OHRQoL severity of our population was higher compared to another study that used the cross‐sectional NHANES 2003–2004 survey of a nationally representative sample of US adults where the mean age was 43 years and the mean severity score was 4.9 (Sanders et al., 2009). Despite the difference in mean age and ethnicity distribution, the most ranked items were associated with function, self‐consciousness and pain and discomfort, which are in accordance with our results. This supports our belief that higher oral health knowledge would relate to positive perception of oral health related quality of life. Participants with poor OHK were about five times more likely to be identified with high levels of severity. Furthermore, overall higher OHK scores were significantly associated with lower OHIP Severity scores in Caucasian and Hispanic participants. It is important to note, that there were 100 Caucasians and 127 Hispanics accounting for 74.7% of the total respondents. Asians comprised only 19.1%, which may be a reason that there was no significant association between OHK scores and OHIP severity.

There are several limitations of the study including the cross‐sectional study design, sampling from a feasibility population that may not properly reflect older adults living in San Bernardino County, and the descriptive nature of the analyses. These constraints mean that caution is required in the interpretation of results. Nevertheless, it can be concluded that it is imperative to develop and implement communication strategies to inform and educate older adults on oral health knowledge with an emphasis on the risk factors associated with periodontal disease and oral cancer. The education should provide equal opportunities for older adults from different ethnicities. It may also be carefully implied that increased oral health knowledge may increase the perception of oral health related quality of life supporting the importance of knowledge delivery to older adults living in San Bernardino County.

## AUTHOR CONTRIBUTION STATEMENT

So Ran Kwon: Conceptualization, Investigation, and Writing; Shirley Lee: Investigation and Writing; Udochukwu Oyoyo: Formal Analysis and Writing; Seth Wiafe: Conceptualization and Writing; Samantha De Guia: Investigation; Caitlin Pedersen: Investigation; Kelsey Martinez: Investigation; Joscelyn Rivas: Investigation; Daniela Chavez: Investigation; Tom Rogers: Writing

## DATA AVAILABILITY STATEMENT

The data that support the findings of this study are available from the corresponding author upon reasonable request.

## CONFLICT OF INTEREST

The authors confirm that they have no conflict of interest to disclose.
